# Technical challenges of intracellular flow cytometry-based assays as a functional complement to diagnosis of signaling defects of inborn errors of immunity: PI3K pathway as a case of study

**DOI:** 10.3389/fimmu.2024.1476218

**Published:** 2024-11-15

**Authors:** Lucía del Pino Molina, Keren Reche Yebra, Yolanda Soto Serrano, Álvaro Clemente Bernal, Carmen L. Avendaño-Monje, J. Gonzalo Ocejo-Vinyals, Rebeca Rodríguez Pena, Eduardo López Granados

**Affiliations:** ^1^ Center for Biomedical Network Research on Rare Diseases (CIBERER U767), Madrid, Spain; ^2^ Lymphocyte Pathophysiology in Immunodeficiencies Group, La Paz Institute for Health Research (IdiPAZ), Madrid, Spain; ^3^ Department of Immunology, Hospital Universitario Central de Asturias, Oviedo, Spain; ^4^ Immunology Department, Hospital Universitario Marqués de Valdecilla, IDIVAL, Santander, Spain; ^5^ Clinical Immunology Department, La Paz University Hospital, Madrid, Spain

**Keywords:** monitoring PI3K-Akt-S6 pathway, inborn errors of immunity (IEI), activated PI3Kδ syndrome (APDS), functional assays, flow cytometry, standardization

## Abstract

**Background:**

The use of next-generation sequencing in inborn errors of immunity (IEI) has considerably increased the identification of novel gene variants, many of which are identified in patients without the described clinical phenotype or with variants of uncertain pathogenic significance in previously described genes. Properly designed functional and cellular assays, many necessarily accomplished by research-based laboratories, reveal the pathogenic consequences of the gene variants and contribute to diagnosis. Activated PI3Kδ syndrome (APDS) is a rare disease that can be divided into APDS1, caused by gain of function (GOF) mutations in *PIK3CD* gene, and APDS2, with loss of function (LOF) variants in the *PIK3R1* gene. Both entities present hyperactivation of the PI3K pathway, which can be analyzed through Akt and S6 phosphorylation status.

**Methods:**

Our objective was to perform an accurate, robust, and reproducible functional assay to analyze the phosphorylation status of proteins in the PI3K-Akt-S6 pathway by flow cytometry, to contribute to diagnosis, to monitor treatments, and to establish intra-assay standardization.

**Results:**

We illustrate the robustness and reproducibility of our experimental procedure in patients with APDS who had high Akt and/or S6 phosphorylation levels at baseline, and after anti-IgM stimulation in B cells. We show the relevance of an appropriate cohort of samples from healthy donors, processed within the same conditions as the suspected samples, in particular the time frame for sample processing once blood is collected.

**Discussion:**

We highlight the importance of B cell stimulation through B cell receptor signaling, which is highly recommended, especially for samples that would be processed more than 24 hours after blood extraction. Also, having a defined experimental procedure is important, including the cytometer setup, which allows cytometer reproducibility for a period of time, enabling the comparison of a sample at different times.

## 1 Introduction

Inborn errors of immunity (IEIs) are a large group of rare diseases caused by pathogenic variants in genes involved in the generation and function of the human immune system (1). The use of next-generation sequencing in clinical practice has accelerated diagnosis, but it has also boosted the identification of patients harboring gene variants of uncertain significance (VUS) or those likely pathogenic, in genes typically associated with clinical and immunological phenotypes that might be coincident or resemble those found in the patient being studied. Functional evaluation of these variants by means of cellular and molecular assays is mandatory for a proper estimation of their pathogenic implications. The type of assays required can exceed the capability of routine laboratories, whereas translational research laboratories can contribute to the diagnosis of IEI with in-house functional assays. However, these assays demand quality control conditions to ensure reproducibility and robustness and should include proper cohorts of healthy controls to account for inter- and intra-individual variation (2).

Activated PI3Kδ syndrome (APDS) is an IEI (3), thus far comprising APDS1, caused by gain of function (GOF) mutations in *PIK3CD* coding for the catalytic p101δ subunit (4, 5); APDS2 with loss of function (LOF) variants in the regulatory *PIK3R1* coding for the regulatory p85α subunit (4) and LOF mutations in phosphatase and tensin homolog (*PTEN*) (APDS-L) (5). These 3 entities promote dysregulation by enhancement of the PI3K-Akt-mTOR-S6 signaling pathway in B and T lymphocytes (6). Hyperactivation of this pathway is associated in B cells with low somatic hypermutation and class switch recombination, which have a profound impact on the germinal center (GC) structure, leading to a reduced memory B-cell compartment and defective humoral response (9), whereas in T cells, the PI3K hyperactivation leads to excessive proliferation and contributes to lymphadenopathy. There is also an enhanced differentiation to memory cells, effector T cells, and T follicular helper, which leads to autoimmunity (7, 8). The CD8 lineage exhibits an exhausted phenotype with effector functions but with impairment of Epstein–Barr virus clearance (9, 10). On the other hand, LOF variants in *PIK3CD* have also been reported leading to underactivation of the PI3Kδ pathway (11, 12).

Single case reports have shown enhanced activity of the pathway by increased basal Akt and S6 phosphorylation levels, downstream of the PI3K, in the primary T and B lymphocytes of patients with APDS. Both western blot and/or phospho-specific intracellular flow cytometry have been used in research-based conditions. We previously reported results suggestive of enhanced Akt and S6 phosphorylation in primary B cells from patients with APDS, both at baseline and after stimulation conditions (4, 13–16). Furthermore, we showed a modulatory effect on compassionate treatment with off-label use of m-TOR inhibition. Increased Akt and S6 phosphorylation levels in T cell blasts, which require a pre-activation culture, have also been reported in patients with APDS. Intracellular flow cytometry is a potentially advantageous technique for the evaluation of signaling pathways in immune cells in a cell-specific manner. However, in contrast to immunophenotyping of leukocyte subpopulations purely based on cell-surface staining, in which standardization has been achieved (17), assays of intracellular flow cytometry remain mostly as research laboratory procedures (18, 19).

We describe a translational research-based, but robust, reproducible and clinically useful in-house functional assay to analyze the phosphorylation status of Akt and S6 in the PI3Kδ pathway by intracellular flow cytometry in primary patient cells. This assay contributes not only to APDS diagnosis (20) but also to the monitoring of PI3K targeted treatments (21–23). We have taken into account several procedural considerations (24) to establish intra-assay standardization through our experimental procedure (21), and a number of processed controls in different periods to reflect the biological variability and establish the normal reference range to compare with suspected APDS cases. We tested positive controls by analyzing baseline and stimulated samples, samples processed in various periods, and importantly, we validated the technique in different clinical and genetically confirmed patients with APDS.

## 2 Materials and methods

### 2.1 Flow cytometer settings and standardization measures

We developed this assay using various flow cytometers. The first studies were performed in a FACS Canto II (BD Biosciences), based on standard day and periodic quality checks to ensure reproducibility. These included setting up the target values for each channel/fluorochrome. In the course of the assay’s development, we were required to change to the use of a DxFlex flow cytometer (Beckman Coulter).

We performed daily quality control checks to ensure that the DxFlex Daily QC Fluorospheres (Beckman Coulter) flow cytometer instrument was working with adequate signal strength and precision. For standardization of the DxFlex flow cytometer, we used Flow-Set Pro fluorospheres (Beckman Coulter). For each specific assay, we defined the target median values or median fluorescence intensities (MFIs), updated daily with Flow-Set Pro fluorospheres, with the adjustment of the optimized gain settings to generate the target median values or MFI.

To achieve comparable results with those from the previous instrument (FACS Canto II), we adapted the MFI from the Canto to the DxFlex, redefining the electronic resolution, because the instruments have different dynamic range (number of channels) (25). We rescaled the target values for each fluorochrome from the Canto to a different scale in the DxFlex by applying a correction factor. We then adjusted the PMT voltages or gains for each fluorochrome in the DxFlex. Once we established those gains or voltages in the DxFlex (comparable to the FACS Canto II instrument), we monitored and updated them daily with the Flow-Set Pro fluorophores, assuming a 5%-10% variation in gains for each fluorochrome. With this methodology, we could compare the results of Akt and S6 phosphorylation from the same patient in the FACS Canto II cytometer and the DxFlex, processed during different periods. The robustness of the assay was enhanced by demonstrating that it can be performed with more than just a single cytometer.

### 2.2 Flow cytometry analysis of intracellular protein phosphorylation levels

We developed a standard operating procedure for an assay (Safe Creative 2408028964461, IMMUNE SIGNAL^®^) that improves the research methodology we previously just applied in two single cases (21, 26). Briefly, fresh peripheral blood mononuclear cells (PBMCs) were obtained after Ficoll density gradient centrifugation, and 5 × 10^5^ PBMCs were resuspended in 500 μl complete medium and transfer to falcon tubes (5 mL) according to the different conditions: unstimulated Akt and S6 to detect basal phosphorylation levels (in independent tubes in triplicates), and stimulated samples for detecting p Akt and p S6 induction after anti IgM activation (in independent tubes in triplicates). The PBMCs were left to rest at 37°C for 30 min and simultaneously stained with surface antibodies: anti-CD27 BV421 (clone M-T271, BD Biosciences) and anti-CD19 PE Cy7 (clone J3-119, Beckman Coulter); we had previously tested the antibodies that could be damaged by methanol solutions for permeabilizing the cells (27). The cells were then fixed at 37°C with prewarmed Lyse/Fix Buffer (Phosflow, BD Biosciences) and permeabilized with Perm III Buffer (Phosflow, BD Biosciences) according to the manufacturer’s instructions. The cells were stained with anti-IgD PE (Southern Biotech) and anti-CD3 APC (clone UCHT1, BD Biosciences), together with the antibodies for detecting phosphorylated residues in targeted proteins: Alexa Fluor 488 (Ser 473) anti-pAkt (clone M9-61, BD Phosflow); Alexa Fluor 488-labeled anti-pS6 (S235-236, clone N7-548, BD Phosflow); and mouse IgG as an isotype control (clone MOPC-21, BD Phosflow). For the version of the assay including activation conditions, PMBCs were stimulated with 15 μg/ml mouse F(ab)2 anti-human IgM (μ chain specific) (Southern Biotech) for 10 min at 37°C, then fixed with Lyse/Fix, proceeding as explained above.

For the version of the assay applied under frozen cell conditions, after the Ficoll procedure, PBMCs were frozen in freezing medium containing 90% fetal calf serum (FCS) and 10% dimethyl sulfoxide (DMSO). The cells were then placed in a cryovial and kept in an isopropanol freezing container at −80°C for 2 days, and then stored in liquid nitrogen. For the thawing process, we placed the cryovial in a water bath at 37°C for some seconds, then added, drop by drop, 1 mL of prewarmed FCS into the cryovial to immediately transfer to 9 mL of prewarmed RPMI. We then centrifuged the cells and resuspended them in RPMI medium to count them, keep in rest for 30 min at 37°C to continue with the protocol previously specified.

For all conditions, the Akt and S6 phosphorylation status was analyzed in different tubes in duplicate or triplicate. Sample acquisition was performed in a DxFlex flow cytometer and analyzed with Flow Jo software.

### 2.3 Samples from patients and healthy donors

Peripheral blood samples from healthy donors (HD) were collected at La Paz University Hospital after obtaining informed consent. Patients with APDS from our clinic or from referring centers were enrolled in the study upon receiving informed consent. The study was approved by the hospital’s ethics committee (PI-4590 and PI-5350) and adhered to the principles set out in the Declaration of Helsinki. Blood samples were collected in lithium-heparin tubes. Depending in the age of the patients, we usually collect for children 2 tubes of 3 ml, while for adults we collect 2 tubes of 8 ml of blood. For travel samples we recommend to send them at room temperature.

The basic information of the genetically-confirmed patients with APDS is provided in **Table 1**. Patient 1 (P1) was a patient with APDS1 previously reported (21) with the mutation c.3061G>A, p. Glu1021>Lys in the *PIK3CD* gene. P3 had both APDS2 and SHORT syndrome due to a c.1425 + 1G>A splicing mutation in the *PIK3R1* gene, as previously reported (26).

**Table 1:** Information of the genetically-confirmed patients with APDS included in this study.

Patient ID	Gene variant	Clinical phenotype	Treatment when functional assay is performed
P1	*PIK3CD* c.3061G>A, p.Glu1021>Lys	APDS1	Sirolimus
P2	*PIK3CD* c.3061G>A p.Glu1021Lys	APDS1	None
P3	*PIK3R1* c.1425 + 1G>A	APDS2; Short syndrome	Sirolimus
P4	*PI3KR1* c.(1425 + 4)_(1425 + 5)del	APDS2; Short syndrome	Sirolimus
P5	*PIK3CD* c.241G>A, p.Glu81Lys	APDS1	None
P6	*PIK3CD*: c.3061G>A p.Glu1021Lys	APDS1	None
P7	*PI3KR1* c.1425 + 2T>G.	APDS2	None

### 2.4 Statistics

The data were analyzed with GraphPad Prism version 9.0 software (San Diego, CA, USA). Statistical differences between patients with APDS and HDs were determined with the Mann–Whitney U test, and for paired samples (fresh vs. frozen) with the Wilcoxon test. P-values <0.05 were considered to have statistical significance and were coded as follows: *p<0.05; **p<0.01; ***p<0.001; ****p<0.0001.

## 3 Results

### 3.1 Impact of the time lapsed from blood extraction to sample processing and analysis

**Figure 1:**
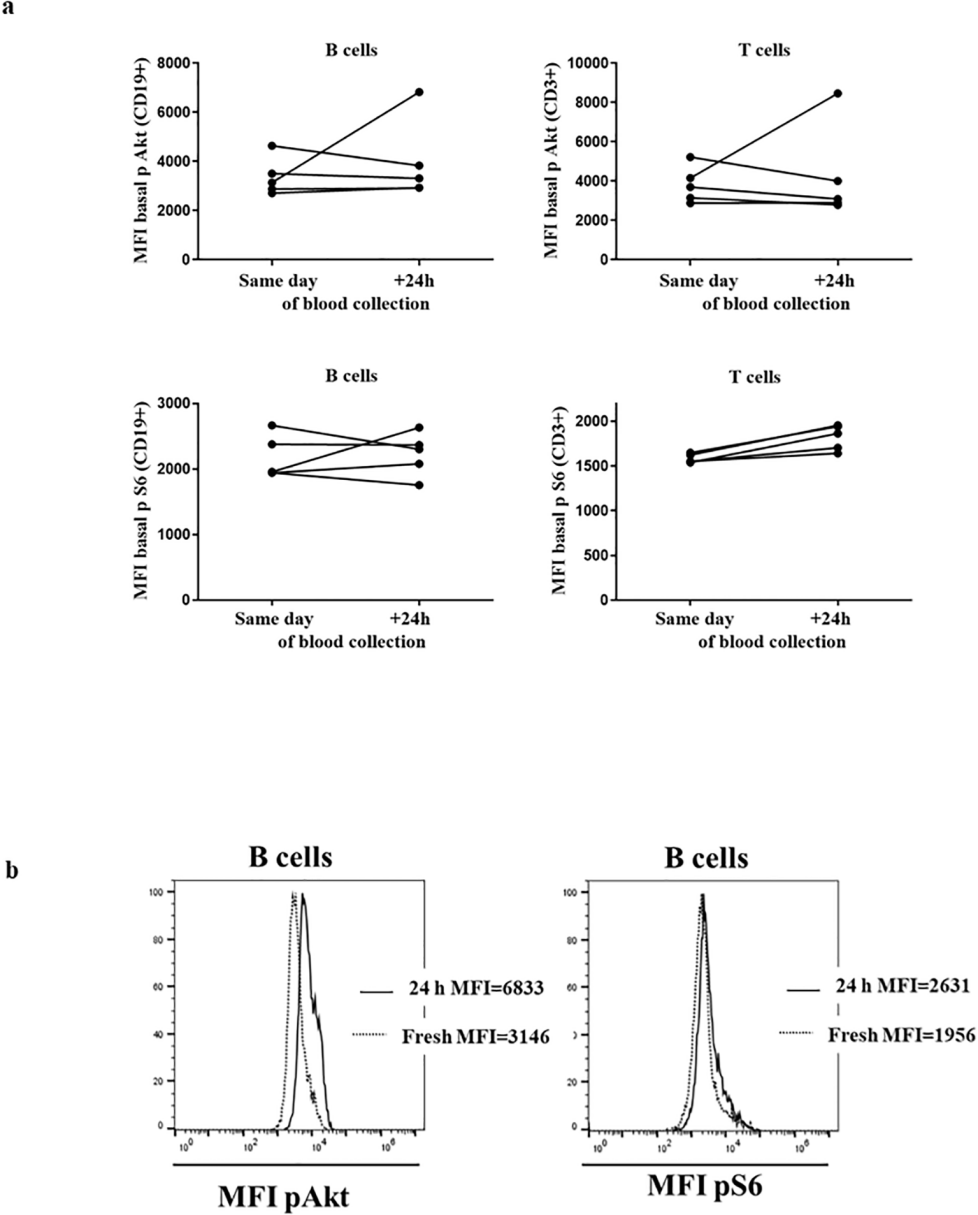
HDs processed on the same day and after 24 h: **(A)** Median fluorescence intensity (MFI) of basal Akt and S6 phosphorylation levels in 3 healthy donors (HDs) in CD19^+^ and CD3^+^ lymphocytes. **(B)** Histograms from representative HD showing the MFI of Akt and S6 phosphorylation in B cells processed same day and after 24 h.

The assay was requested as a complementary evaluation of gene variants in suspected APDS cases from other centers. Therefore, we anticipated the need for analyzing fresh samples that had traveled for some time from the time of extraction. First, we compared the performance of the assay in blood collection samples (travel samples) obtained from healthy donors (HDs) processed on the same day (SD) vs. those processed 24 hours later (next day [ND]). In these samples, we observed slight differences in unstimulated conditions between the mean MFI of pAkt and pS6 in total CD19^+^ and CD3^+^ lymphocytes (**Figures 1A, B**). Therefore, we considered it convenient to establish 2 sets of results from HDs, SD or ND, to be introduced into the assay as normal reference values for evaluation, **Supplementary Table S1**. The results from the HDs processed in parallel at 2 time points were included in the corresponding HD cohort.

### 3.2 Establishment of inter-assay variability and normal ranges for same day and next day assays

**Figure 2:**
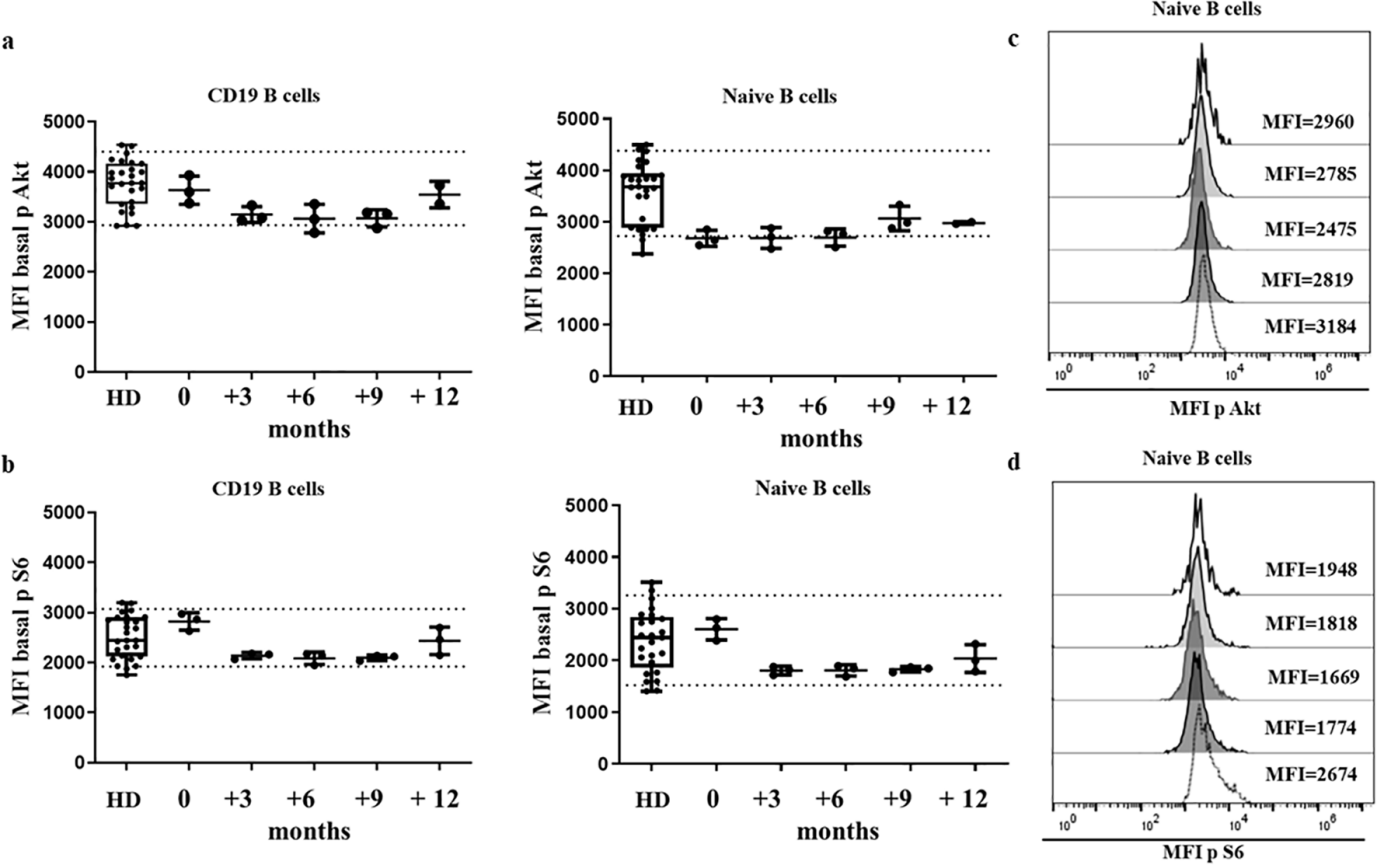
HD stability of sample over time. Median fluorescence intensity (MFI) of Akt **(A)** and S6 **(B)** basal phosphorylation levels of healthy donors (HDs) and one HD in which the assay was run in triplicate, on 5 different occasions over a 12-month period. Dotted lines indicate the 10^th^ and 90^th^ percentile of HDs. Histogram from the HD with Akt **(C)** and S6 **(D)** phosphorylation levels represented as MFI in 5 different occasions.

The establishment of normal ranges in HDs requires controlling the intrinsic inter-assay variability by means of a daily quality control check, and the update of cytometer settings, enabling the accumulation of results from HDs processed on different days. Similar values were obtained by triplicates from the same donor, processed the ND from separated blood samples over 1 year of follow-up, as shown in **Figures 2A–D**. These results indicate that our experimental procedure ensures reproducibility.

For both sets of HDs, processed the SD or ND, we defined a normal reference MFI range by the 10^th^ and 90^th^ percentile of the cumulative results. Normal ranges in box-plot figures are shown in the corresponding figures in the manuscript and in **Supplementary Table S1**. We gradually increased the size of the normal cohorts by processing an HD sample in parallel every time a functional assay of baseline Akt and S6 phosphorylation was performed in patients with suspicion of APDS or who carried variants in *PIK3CD* or *PIK3R1*. So far, 30 HDs processed the SD as blood extraction and 20 HDs processed the ND after blood extraction account for the normal ranges of baseline Akt and S6 phosphorylation levels. Duplicates or triplicates were included for each sample to attenuate intra-assay variability.

As a complementary analysis, normal ranges of Akt and S6 phosphorylation were also established in stimulated conditions upon anti-IgM incubation in samples analyzed in duplicate from 11 HDs processed the SD and 9 HDs processed the ND after blood extraction. Normal ranges in box-plot figures are shown in the corresponding figures in the manuscript.

### 3.3 Assay performance in confirmed activated PI3K delta syndrome cases processed the same day as blood extraction

We validated the capacity of our assay to discriminate differences in the phosphorylation of targeted proteins in primary B cells from fresh samples processed the SD, between the range of normal controls and genetically confirmed patients with APDS. Four patients with APDS1 and confirmed variants in *PIK3CD* (P1, P2, P5 and P6) and 3 patients with APDS2 and SHORT syndrome harboring variants in *PI3KR1* (P3, P4 and P7) were included.

The GOF effect for the PI3K pathway of gene variants from P1 and P3 had been suggested in our previous reports, by the increased baseline phosphorylation status of Akt and/or S6 in patient B cells compared with a single HD in samples processed the SD as extraction (21, 26). However, the lack of standardization methods and comparative results from a representative cohort of HDs precluded us to establish firm estimations at that time.

**Figure 3:**
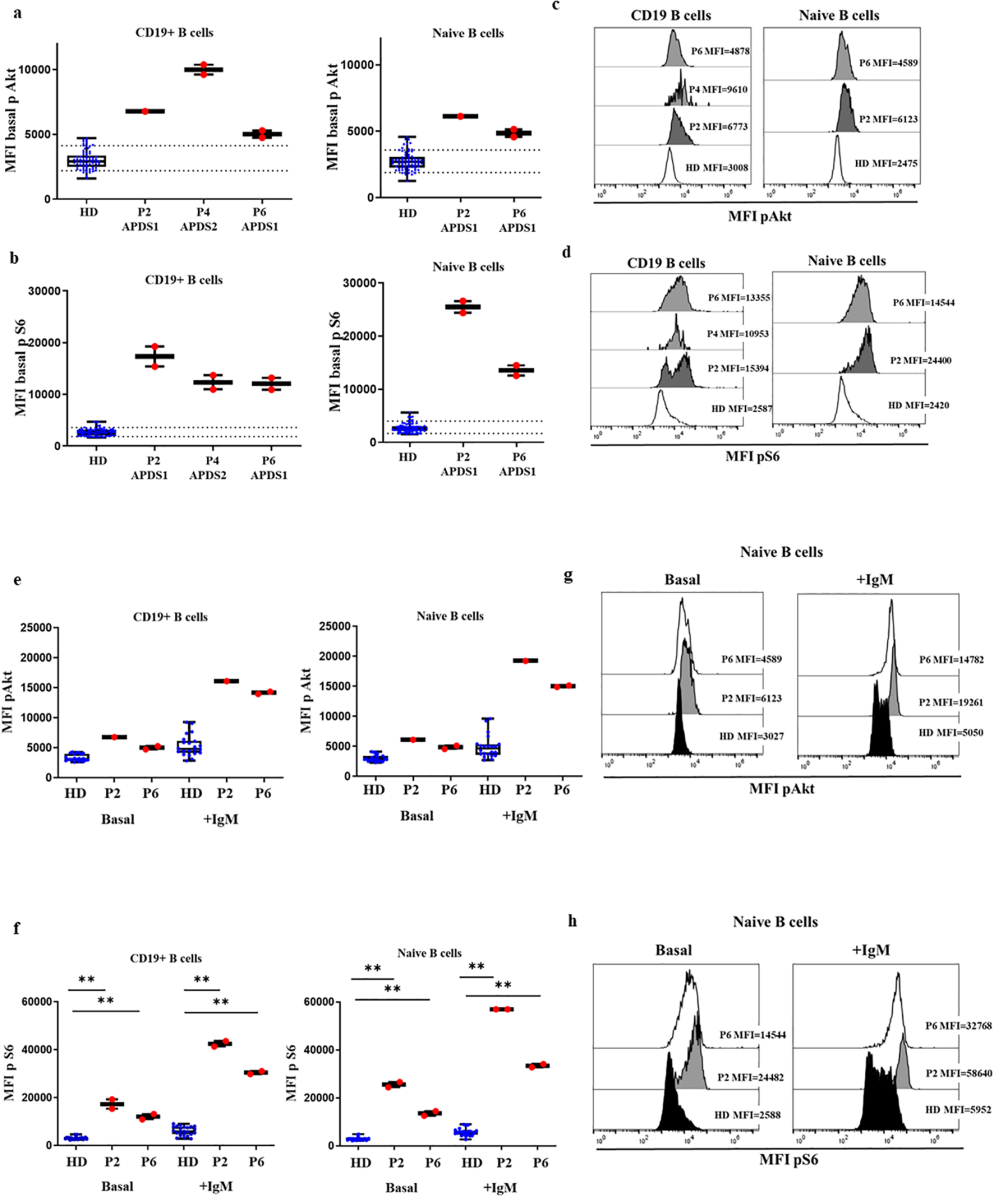
Confirmed cases APDS1 and APDS2 in DxFlex processed on the same day of blood extraction. Median fluorescence intensity (MFI) of Akt **(A)** and S6 **(B)** basal phosphorylation levels in B cells and naïve B cells, from 30 HDs processed on the same day as blood extraction, P2, P4 and P6. Samples analyzed in a DxFlex flow cytometer. Dotted lines indicate the 10^th^ and 90^th^ percentile of HD. Representative MFI kinetics of Akt phosphorylation **(C)** and S6 phosphorylation **(D)** in B cells and naïve B cells for representative HDs and patient P2 (APDS1), P4 (APDS2) and P6 (APDS1). Median fluorescence intensity (MFI) at basaline and after stimulation with anti-IgM for 10 minutes of Akt **(E)** and S6 **(F)** phosphorylation levels in B cells and naïve B cells. Representative MFI kinetics of Akt phosphorylation **(G)** and S6 phosphorylation **(H)** in naïve B cells in unstimulated and anti-IgM conditions for representative HDs and patients P2 and P6. **p<0.01.

We then explored the performance of the assay of patients with APDS1 and APDS2 in SD conditions. As shown in **Figures 3A–D**, P2 and P6 presented remarkably higher baseline phosphorylation levels of mean triplicate values for S6 in CD19^+^ B cells (MFI P2 = 17328, MFI P6 = 12047 vs HD mean MFI=2647 [90^th^ percentile=3584]), which was even higher in naïve B cells (MFI P2 = 25512, MFI P6 = 13526 vs HD mean MFI=2532 [90^th^ percentile=3918]). In panel 3d, a bimodal distribution for increased S6 phosphorylation is appreciated for P2, corresponding lower and higher MFI to memory and naïve B cells respectively (**Supplementary Figure S1**). Phosphorylation levels of Akt in P2 and P6 were well over the 90^th^ percentile of HD for total B cells (MFI P2 = 6773, MFI P6 = 5016 vs HD mean MFI=2912 [90^th^ percentile=4162]) and naïve B cells (MFI P2 = 6123, MFI P6 = 4857 vs HD mean MFI=2685 [90^th^ percentile=3677]). Given that P4 presented a severe reduction of total B cells, the analysis of baseline phosphorylation could only be performed for total B cells to collect a representative number of events, without discriminating the particular status in naïve B cells. Baseline phosphorylation levels were greatly higher for both Akt and S6 compared with HDs (MFI=11261 vs. 2912 for Akt; MFI=12320 vs. 2647 for S6). Baseline phosphorylation levels to detect the functional gain of function impact of gene variants should be compared in naïve B cells, to ensure homogeneity in terms of similar cell composition in HD and different APDS patients.

We also analyzed the induction of phosphorylation after anti-IgM activation of naïve and unswitched memory B cells in P2 and P6. In those patients, total B cells and naïve B cells presented higher levels of phosphorylated Akt (MFI P2 = 16117, MFI P6 = 14167 vs. HD mean MFI=4819 in total B cells and in naïve B cells MFI P2 = 19261, MFI P6 = 15025 vs. HD mean MFI=4658, respectively) and S6 after anti-IgM activation compared with 11 controls (MFI P2 = 42487, MFI P6 = 30465 vs. HD mean MFI=5903 and MFI P2 = 56980, MFI P6 = 33447 vs. HD mean MFI=5668, respectively), (**Figures 3E–H**).

### 3.4 Assay performance in confirmed activated PI3K delta syndrome cases processed the day after blood extraction

**Figure 4:**
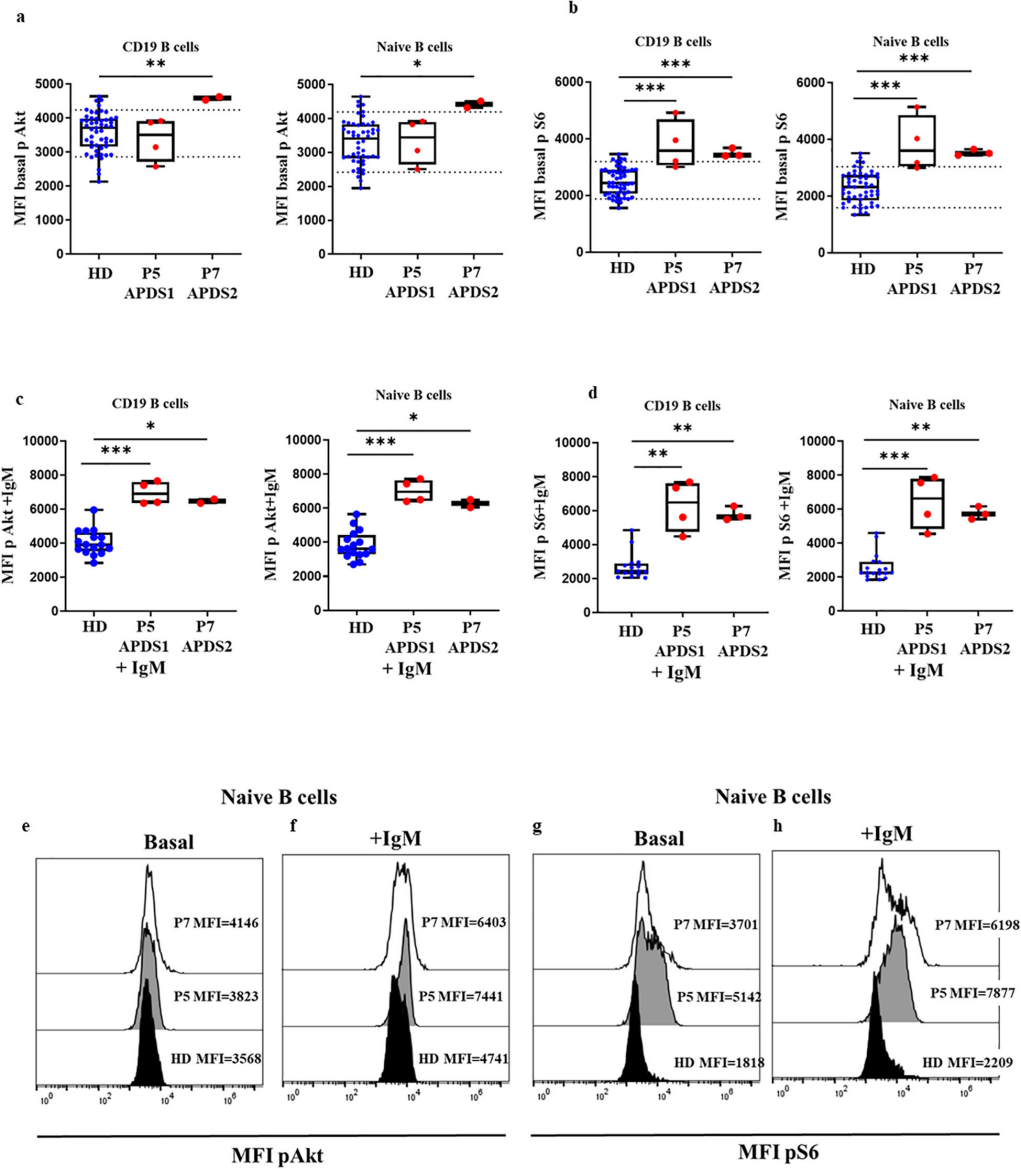
Confirmed APDS1 and APDS2 cases processed 24 h after blood extraction. Median fluorescence intensity (MFI) of basal phosphorylation levels of Akt **(A)** and S6 **(B)** in B cells and naïve B cells of HDs, P5 and P7 processed 24 hours after blood extraction. Phosphorylation induction of Akt **(C)** and S6 **(D)** with anti-IgM activation in B cells and naïve B cells. Representative histograms from P5 showing phosphorylation levels in naïve B cells at baseline and after anti-IgM activation of Akt **(E, F)** and S6 **(G, H)** processed 24 hours after blood extraction, *p<0.05; **p<0.01; ***p<0.001.

We received two patients sample P5 and P7 from two referring centers that were analyzed in triplicate together with a travel sample and a local 24 h extracted sample from an HD. In P5, we found baseline Akt phosphorylation levels (**Figure 4A**) in total B cells similar to those of the HD, and a slightly increased levels in P7 (MFI P5 = 3508, MFI P7 = 4581 vs. mean MFI HD=3716 (P7, p=0.065) and similar results in naïve B cells (MFI P5 = 3440, MFI P7 = 4417 vs. MFI HD=3406, respectively, P7 p=0.065) (**Figure 4B**). While the phosphorylated S6 levels of P5 and P7 were in the high range of the HD in total B cells (MFI P5 = 3580, MFI P7 = 3412 vs. mean MFI HD=2436, p=0.0008 and p=0.0003); and in naïve B cells, MFI P5 = 3607, MFI P7 = 3510 vs. mean MFI HD=2329, p=0.0005 and p=0.0002). These results suggested that baseline hyperphosphorylation can be affected in ND samples by time elapsed from blood extraction and sample processing. We confirmed these results with 2 independent samples collected at 2 different follow-up visits (**Figures 4A, B, E–G**).

**Figure 5:**
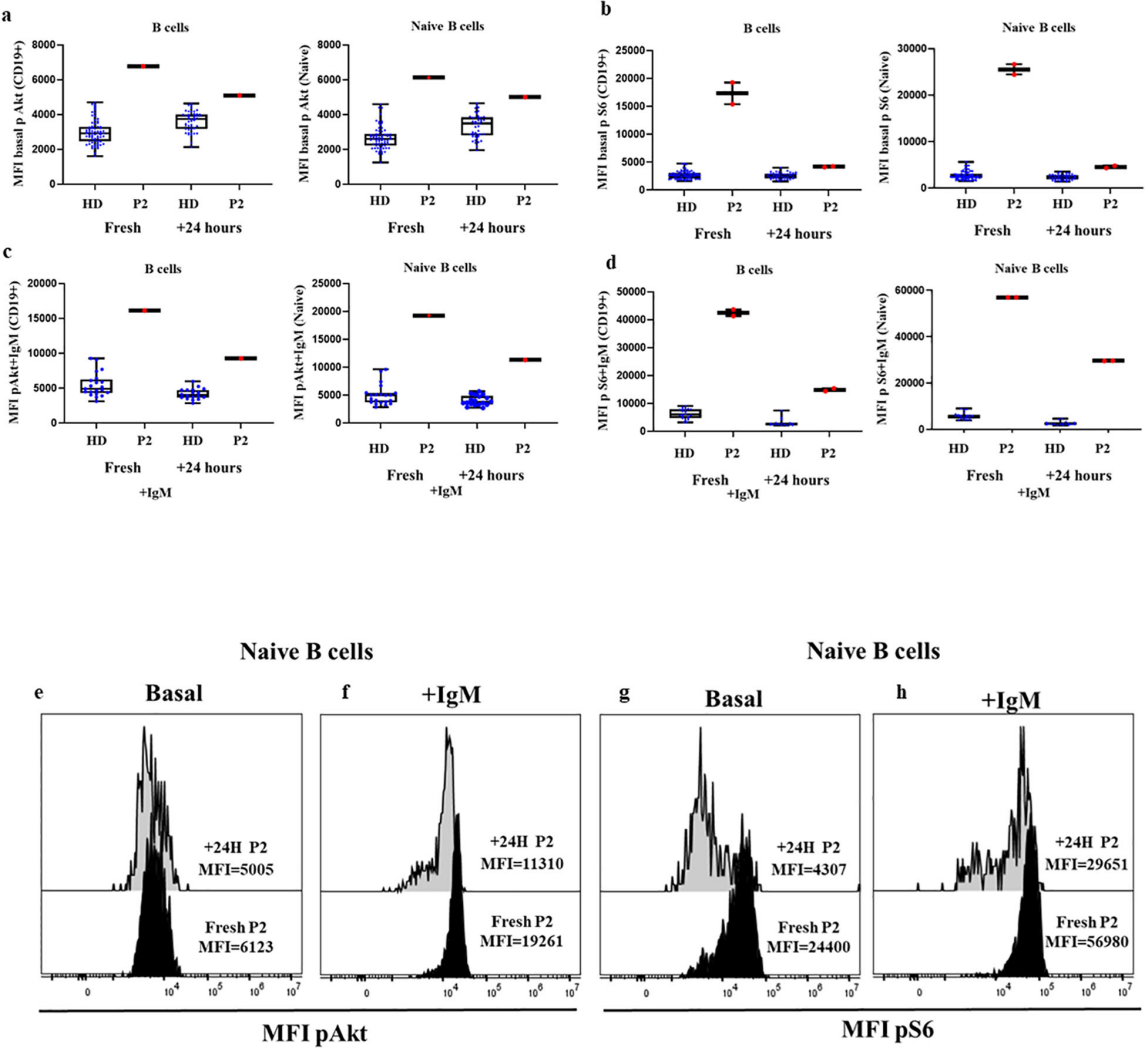
Comparison of confirmed APDS1 case processed the same day as blood extraction and 24 hours later at basal and after anti-IgM activation. **(A)** Median fluorescence intensity (MFI) of basal Akt **(A)** and S6 **(B)** phosphorylation levels in B cells and naïve B cells from HDs and P2 processed the same day as blood extraction or after 24 hours. Phosphorylation induction with anti-IgM activation of Akt **(C)** and S6 **(D)** in B cells and Naïve B cells. Representative histograms from P2 showing the comparative phosphorylation levels in naïve B cells at basal and after anti-IgM activation of Akt **(E, F)** and S6 **(G, H)** processed same day or 24 h after blood extraction.

To assess whether enhanced Akt and S6 phosphorylation levels could be detected by flow cytometry in those fresh samples processed 24 hours after blood extraction, we processed new samples from P2 on the same day of blood extraction and 24 hours later. We found a slight reduction in MFI in relation to Akt phosphorylation levels in total B cells and naïve B cells (6773 vs. 5095; 6123 vs. 5005, respectively) and for S6 (17328 vs 4184; 25512 vs. 4547, respectively), although in both cases the results were much higher than in the HD cohort, respectively processed the SD of blood extraction or ND 24 hours after (**Figures 5A, B, E–G**). Also, there was a reduction in phosphorylation levels of Akt in total B cells and naïve B cells after anti-IgM activation 24 hours after blood extraction (16117 vs. 9252; 19261 vs. 11310, respectively) and in S6 (42487 vs. 14858; 56980 vs. 29601, respectively), but it remained higher than in the corresponding HDs (**Figures 5C, D, F–H**).

In P5 and P7 after activation with IgM, we found statistically significantly higher levels of phosphorylation in total B cells compared with 9 HDs for Akt (MFI P5 = 6907, MFI P7 = 6470 vs. MFI HD=3909, p=0.0004 and p=0.0131, respectively), and naïve B cells (MFI P5 = 6975, MFI P7 = 6279 vs. MFI HD=3612, p=0.0004 and p=0.0131, respectively) (**Figures 4C, F**). And also higher phosphorylation levels of S6 in total B cells (MFI P5 = 6487, MFI P7 = 5646 vs. MFI HD= 2435, p=0.0008 and p=0.0021) and in naïve B cells MFI P5 = 6639, MFI P7 = 5715 vs. MFI HD=2273, p=0.0005 and p=0.0015, respectively) (**Figures 4C, H**).

These results suggest that to demonstrate a GOF of the mutated proteins by enhanced activity of the PI3K signaling pathway by means of demonstrating enhanced phosphorylation of AKT and S6, pre-activation to achieve maximum activity is required.

### 3.5 Performance of the assay for monitoring down-modulation of the PI3K-Akt-mTOR S6 pathway in patients with activated PI3K delta syndrome treated with mTOR inhibitors

We had previously reported that in P1 treatment with sirolimus, an mTOR inhibitor that attenuates hyperactivation of the PI3K-Akt-mTOR-S6 pathway, had decreased baseline phosphorylation levels for Akt and S6 compared with pre-treatment levels (21).

P1 (APDS1) had continued treatment by the time our assay was properly implemented, and P3 (APDS2) had initiated treatment with sirolimus after the baseline study had been performed, also with the validated assay by the time we used a FACS Canto II. Now, we again analyzed the phosphorylation status in fresh B cells from P1 and P3.

**Figure 6:**
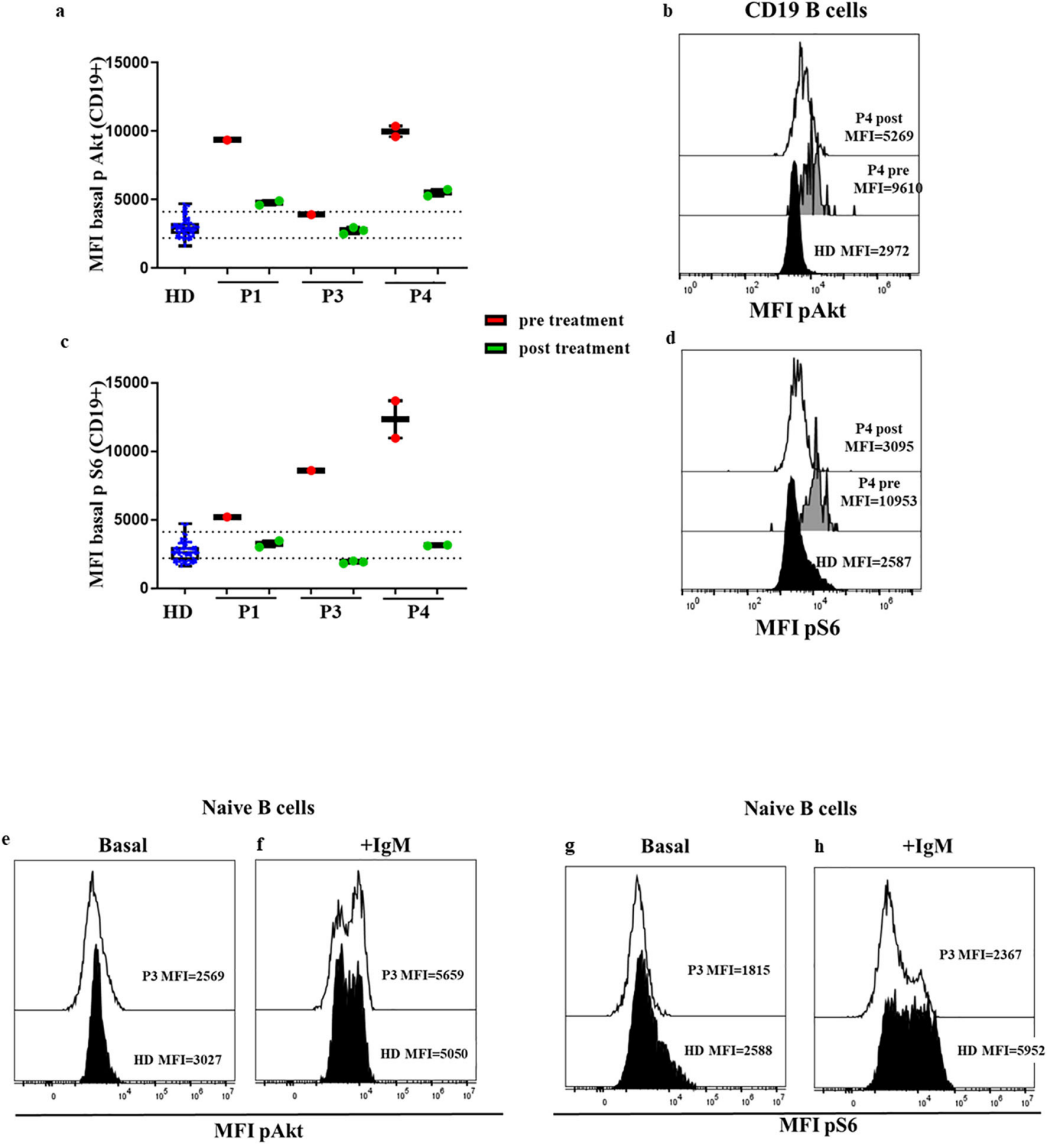
Confirmed APDS1 and APDS2 cases in treatment with sirolimus. Median fluorescence intensity (MFI) of basal Akt **(A)** and S6 **(B)** phosphorylation levels in B cells from HDs processed on the same day of blood extraction; P1 (APDS1), P3, and P4 (APDS2) phosphorylation levels pre- and post-treatment with sirolimus are shown. Dotted lines indicate the 10^th^ and 90^th^ percentile of HDs. Representative MFI kinetics of Akt phosphorylation **(C)** and S6 phosphorylation **(D)** in B cells for representative HDs and P4 (APDS2) pre- and post-treatment with sirolimus. Comparable results of basal Akt and S6 phosphorylation in B cells from patient P1 and P3 at diagnosis in a FACS Canto II flow cytometer, and some years later with sirolimus treatment in a DxFlex flow cytometer. Results are expressed at the dynamic range of FACS Canto II. Representative histograms of Akt **(E, F)** and S6 phosphorylation **(G, H)** in P3 during treatment with sirolimus at basaline and after anti IgM activation in naïve B cells.

We found baseline phosphorylation levels of Akt in B cells (P1 MFI=4763, P3 MFI=2762 vs. mean MFI HD=2912 [percentile 2201-4162]) and naïve B cells (P1 MFI=4389, P3 MFI= 2635 vs. mean MFI HD=2685 [percentile 1938-3677]) (**Figures 6A, B**) almost within the normal range of HDs while the patients were under sirolimus. We obtained similar results for S6 phosphorylation in B cells (P1 MFI=3238, P3 MFI=1905 vs. mean MFI HD=2647 [percentile 1847-3584]) and naïve B cells (P1 MFI=3058, P3 MFI=1818 vs. mean MFI HD=2532 [percentile 1725-3918]), (**Figures 6C, D**). In P3 we also analyzed the phosphorylation levels after anti IgM during sirolimus treatment, and we found similar levels of Akt and S6 to those of HD (**Figures 6E–H**).

Moreover, the standardization allowed us to compare original results before treatment was initiated from patient P3 (26) analyzed in a FACS Canto II flow cytometer, with new results some years later while the patient was stable on sirolimus, in a DxFlex flow cytometer. We detected the attenuation of previously increased baseline S6 phosphorylation levels in B cells (MFI=541 vs. 119). We also monitored the response to sirolimus in P4, and the functional assay showed the reduction of baseline Akt (11261 vs. 5504) and S6 phosphorylation (12320 vs. 3127) in total B cells after treatment (**Figures 6A–D**).

### 3.6 Impact of the use of fresh vs. frozen cells in the assay capacity to reveal enhanced activity of the pathway in activated PI3K delta syndrome-confirmed patients

**Figure 7:**
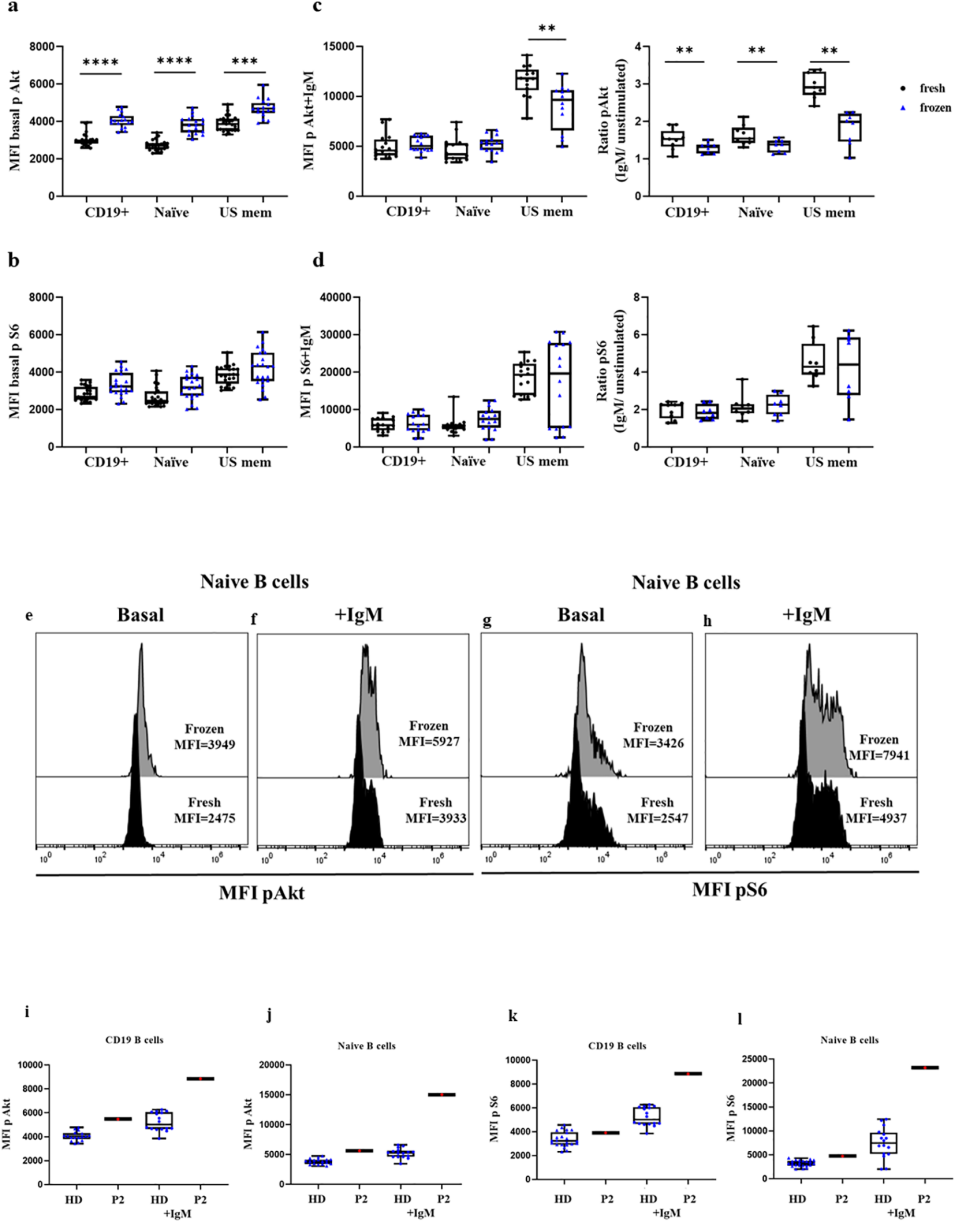
Comparison of Akt and S6 phosphorylation levels in fresh vs. frozen PBMCs. Median fluorescence intensity (MFI) of basal Akt and S6 phosphorylation levels in B cells, naïve, and unswitched memory B cells (US mem) from fresh PBMCs of HDs (in black) or frozen PBMCs (in blue) in unstimulated **(A, B)** and stimulated conditions **(C, D)**. Ratio of Akt and S6 phosphorylation induction after anti-IgM activation. Representative histograms of HDs showing phosphorylation levels of Akt **(E, F)** and S6 **(G, H)** in naïve B cells at basal conditions and after anti-IgM activation in fresh and frozen samples. Comparison between frozen PBMCs from HDs and P2 in total CD19^+^ B cells and naïve B cells at baseline and after anti-IgM stimulation conditions for Akt **(I, J)** and S6 **(K, L)**; *p<0.05; **p<0.01; ***p<0.001; ****p<0.0001.

Lastly, we compared the phosphorylation levels in HDs processed the same day as blood extraction in fresh samples and the same frozen samples. We analyzed the baseline phosphorylation of Akt and S6 in a total of 11 HDs, and after anti IgM activation in 9 HDs. In the HDs we found slightly higher baseline phosphorylation levels in frozen samples of Akt in total B cells (4040 vs. 2954, p<0.0001), naïve cells (3818 vs. 2707, p<0.0001), and unswitched memory B cells (4680 vs. 3854, p=0.0001), as well as for S6 phosphorylation in frozen vs. fresh samples in total B cells (3224 vs. 2663, p=0.0029), naïve B cells (3170 vs. 2445, p=0.0050), and unswitched memory B cells (4336 vs. 3870) (**Figures 7A, B, E, G**). Both unswitched memory B cells and naïve B cells in fresh and frozen samples reached similar phosphorylation levels in Akt after stimulation with anti-IgM (5023 vs. 4572 in total B cells, and 5269 vs. 4182 in naïve B cells, p=0.04) and for S6 (6158 vs. 5844 in total B cells and 7973 vs. 5399 in naïve B cells, p=0.0186) (**Figures 7C, D, F, H**). However, the ratio for Akt in stimulated cells vs. unstimulated samples was slightly lower in the frozen samples compared with the fresh ones (p=0.0294). We also evaluated the phosphorylation levels in frozen PBMCs from P2. In P2 we observed a slight increment in the phosphorylation levels of baseline Akt and S6 in naïve B cells, and the hyperactivation of the pathway was more evident after anti-IgM activation (**Figures 7I–L**). The phosphorylation levels of frozen samples from P2 were similar to those obtained in fresh sample from the same patient 24 hours after blood extraction.

## 4 Discussion

Flow cytometry is a pivotal routine technology for the diagnosis of IEI (28). Its mainstream application is the phenotypic evaluation and enumeration of immune cells based on cell surface protein detection. This is accomplished by applying protocols that are relatively low in cost, reproducible, sensitive, an easy-to-use with current platforms, allowing complete analysis and data interpretation in a short period (28, 29). In addition, standardized procedures and protocols have been developed that allow for data comparison and integration in collaborative multicenter initiatives (17, 30).

Flow cytometry can also be employed for the detection of intracellular disease-related proteins (31), whose level of expression can be directly affected by gene variants, or to detect changes in protein status or phosphorylation along signaling pathways, as read-out of cell function assays to reveal the functional impact of gene variants on proteins upstream of the evaluated event.

Protocols for intracellular detection are considerably more difficult and prone to variability, which can affect various parameters. Functional assays based on cell activation in various *ex vivo* or *in  vitro* conditions, followed by intracellular protein detection, remain as in-house research-grade laboratory procedures. They are performed sporadically, with patients’ cells typically only compared with same-day healthy controls.

However, proper attention to critical parameters is intrinsic to these assays: the time frame for sample processing, optimization of sample preparation with fixation and permeabilization steps to simultaneously detect surface and intracellular targets, the use of positive controls, the establishment of appropriate cut-offs based on a sufficient number of normal controls, and a correct instrumental setup to account for inter-assay variability. All these steps allow for the use of these assays as a complement to routine diagnosis from laboratories in highly specialized centers (28, 29).

Our research group has broad experience in the immune cell signaling assessment by intracellular cytometry (IMMUNE SIGNAL^®^), and we are familiar with the principles of standardization and quality control in routine diagnostic settings. Therefore, we implemented a number of in-house research-based assays developed under reproducible conditions that account for individual, intra-, and inter-assay variability, ensuring their usefulness for the clinical interpretation of the results.

APDS disease comprises 3 distinct entities, APDS1, APDS2, and LOF mutations in PTEN (APDS-L) (5). In all cases, there is hyperactivation of the PI3K signaling pathway, which can be inferred through Akt and S6 phosphorylation, two downstream proteins, in lymphocytes. To detect the enhanced Akt and S6 phosphorylation, functional assays have occasionally been reported to have been applied to cells from patients with APDS, based on intracellular flow cytometry or western blot (13–15, 32, 33).

Well-established pathogenic variants enhance Akt and/or S6 phosphorylation in primary B and T cells. Other studies have also reflected the relevance of increased Akt and S6 phosphorylation in T cell blasts as an informative functional test for APDS confirmation; however, this involves a longer process including maintaining T cells in culture for several days, which is even more prone to intrinsic variability than primary unstimulated cell analysis or a short-term activation-based experiment (33). Only robust and reproducible functional assays should be applied for new pathogenic variants or VUS, in which it is necessary to establish the casual relationship between genotype and phenotype (2).

We illustrated the robustness and reproducibility of our assay in patients with APDS, with high baseline Akt and/or S6 phosphorylation levels and after anti-IgM stimulation in B cells. We showed the relevance of including an appropriate cohort of HDs processed within the same conditions as that of suspected samples, especially the time elapsed for processing the sample from when the blood is collected. In a patient with APDS (P2) who had a described variant, we compared the behavior of Akt and S6 phosphorylation on the same day of blood extraction and 24 hours later, detecting a reduction in baseline Akt and S6 phosphorylation levels in B cells at 24 hours. Our results were similar to those of Angulo et al., who reported the degradation of protein and the impossibility of performing a western blot to detect the phosphorylation status (14). Angulo et al. suggested increased cell death due to hyperactivation of the pathway. We could not formally prove this in our experimental procedure; however, we consistently detected higher phosphorylation levels of Akt and S6 in baseline conditions without activation in fresh samples from patients with relevant pathway hyperactivation.

We highlight the importance of B cell stimulation, through B cell receptor signaling, which is highly recommended, especially for samples that would be processed 24 hours after of blood extraction and that are studied to assess a causal relationship between the genetic variant and the functional effect.

In addition, it is important to consider an assay as complementary to routine practice. A well-defined experimental procedure and appropriate cytometer set up is key, which would allow the intra-assay to be performed to the same standard as the laboratory. In this way, we could progressively accumulate healthy control participants, leading to a good representation of biological variation among individuals and establishing an accurate cutoff within the 10^th^-90^th^ percentile.

Moreover, cytometer reproducibility for a given period enables the comparison of a sample at different times. For example, we could compare samples from patients at diagnosis and during monitored treatments to ameliorate clinical symptoms in those with APDS, and, in particular, lymphoproliferation (34). We and others have also monitored the phosphorylation levels of Akt and S6 after PI3K inhibitor treatments (21–23, 35).

After carefully taking into consideration all the above aspects, our functional assay is robust by confirming the hyperactivation of the PI3K pathway in already described variants. And to test the pathogenicity by analyzing this pathway in novel non-described variants. Thus, patients could benefit from treatments that are more precise and that can be monitored over time. Moreover, this functional assay could be implemented for other IEI, but focusing on T cells to test variants in other primary immunodeficiency-related genes, with dysregulation of the PI3K-Akt-S6 pathway as previously described in some variants of CARD11 with reduced S6 phosphorylation (36), or enhanced Akt phosphorylation in double negative T cells from autoimmune lymphoproliferative syndrome (37).

However, there are still some limitations; for example, patients with APDS presented higher numbers of transitional B cells (38), which might also involve a higher Akt phosphorylation level (15). Also, for pediatric patients, an age-matched HD cohort should be included. For the less frequent, but possible LOF variants in *PIK3CD* in which there is a severe reduction of B cells, the appropriate evaluation of the reduced T cell phosphorylation with specific stimuli would be also informative (39).

There was no major difference in Akt and S6 induction in frozen PBMCs, although the baseline phosphorylation levels were upregulated in frozen PBMCs compared with the same blood extraction samples collected and processed on the same day. Asano et al. also observed that frozen samples from patients with APDS presented higher phosphorylation levels of Akt than that of the HDs (15). Based on a sample from the patient with APDS1 our results indicate that hyperphosphorylation of Akt and/or S6 could be detected at baseline and after stimulation in frozen samples. However, frozen samples from suspected APDS patients should be carefully processed in the same freezing and thawing conditions than controls.

The challenge remains for all the VUS found in patients, which are difficult to categorize clinically, or in patients with concomitant complications undergoing treatments that might attenuate the immune system and could interfere with the identification of hyperactivation in this pathway. To summarize, we showed the robustness of our experimental procedure to detect hyperactivation of the PI3K pathway, with confirmed cases of APDS. We recommend to evaluate the activity of this pathway in patients with suspected APDS, preferentially in fresh samples, in basal and after activation conditions, especially for samples processed more than 24 hours after blood extraction.

## Data Availability

The original contributions presented in the study are included in the article/**Supplementary Material**. Further inquiries can be directed to the corresponding author.

## Supplementary material

SUPPLEMENTARY TABLE 1 Data sheet of HD showing median MFI, maximum, minimum and percentiles 10th and 90th, for Akt and S6 phosphorylation levels processed same day (SD) or next day (ND) of blood extraction at baseline and after anti IgM activation.
