# Supplementary material for: Technical challenges of intracellular flow cytometry-based assays as a functional complement to diagnosis of signaling defects of inborn errors of immunity: PI3K pathway as a case of study
Source: Front Immunol. 2024 Nov 15;15:1476218. doi: 10.3389/fimmu.2024.1476218 (PMC11604744; doi:10.3389/fimmu.2024.1476218)
Supplement: SUPPLEMENTARY TABLE 1 — Data sheet of HD showing median MFI, maximum, minimum and percentiles 10th and 90th, for Akt and S6 phosphorylation levels processed same day (SD) or next day (ND) of blood extraction at baseline and after anti IgM activation. [file DataSheet1.pdf]

Supplementary Table 1:

	HD processed same day blood extraction (SD)			
	MFI baseline p Akt		MFI baseline p S6	
	CD19 B cells	Naive B cells	CD19 B cells	Naive B cells
**Median**	2912	2685	2647	2532
**Minimum**	1603	1256	1609	1520
**10% Percentile**	2201	1938	1847	1725
**90% Percentile**	4162	3677	3584	3918
**Maximum**	4703	4589	4722	5623

	HD processed next day blood extraction (ND)			
	MFI baseline p Akt		MFI baseline p S6	
	CD19 B cells	Naive B cells	CD19 B cells	Naive B cells
**Median**	3716	3406	2436	2329
**Minimum**	2130	1948	1558	1341
**10% Percentile**	2862	2420	1878	1591
**90% Percentile**	4241	4194	3193	3047
**Maximum**	4635	4648	3451	3510

	HD processed same day blood extraction (SD)			
	p Akt induction with IgM		p S6 induction with IgM	
	CD19 B cells	Naive B cells	CD19 B cells	Naive B cells
**Median**	4819	4658	5903	5668
**Minimum**	2842	2729	2819	2685
**10% Percentile**	3295	2972	3173	3999
**90% Percentile**	8662	8710	8390	8646
**Maximum**	9266	9611	9080	8993

	HD processed next day blood extraction (ND)			
	p Akt induction with IgM		p S6 induction with IgM	
	CD19 B cells	Naive B cells	CD19 B cells	Naive B cells
**Median**	3909	3612	2435	2273
**Minimum**	2842	2707	2054	1848
**10% Percentile**	3155	2793	2077	1862
**90% Percentile**	5118	5279	4372	4411
**Maximum**	5952	5646	4858	4608
