# Supplementary figures and images for: Technical challenges of intracellular flow cytometry-based assays as a functional complement to diagnosis of signaling defects of inborn errors of immunity: PI3K pathway as a case of study
Source: Front Immunol. 2024 Nov 15;15:1476218. doi: 10.3389/fimmu.2024.1476218 (PMC11604744; doi:10.3389/fimmu.2024.1476218)

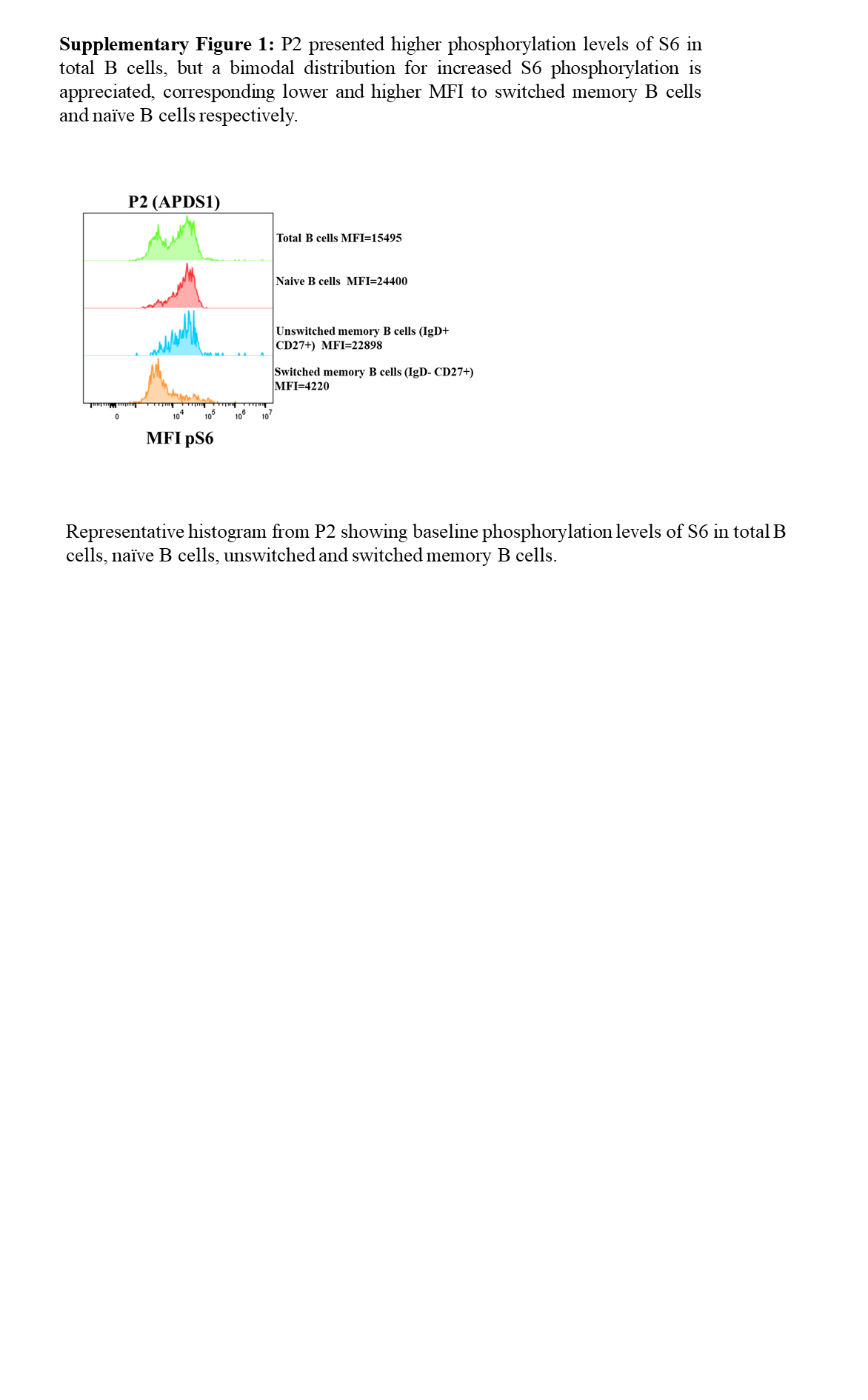

Supplement: Supplementary file 2 [file Image1.tif]
